# Nonmicrobial mechanisms dominate the release of CO_2_ and the decomposition of organic matter during the short-term redox process in paddy soil slurry

**DOI:** 10.1016/j.eehl.2023.08.005

**Published:** 2023-08-29

**Authors:** Jinsong Liu, Changyin Zhu, Xiantang Liu, Xiaolei Wang, Dongmei Zhou

**Affiliations:** State Key Laboratory of Pollution Control and Resource Reuse, School of the Environment, Nanjing University, Nanjing 210023, China

**Keywords:** Soil, Dissolved organic matter, Abiotically mediated CO_2_ emission, Reactive oxygen species

## Abstract

Both biotic and abiotic mechanisms play a role in soil CO_2_ emission processes. However, abiotically mediated CO_2_ emission and the role of reactive oxygen species are still poorly understood in paddy soil. This study revealed that •OH promoted CO_2_ emission in paddy soil slurries during short-term oxidation (4 h). •OH generation was highly hinged on active Fe(II) content, and the •OH contribution to CO_2_ efflux was 10%–33% in topsoil and 40%–77% in deep-soil slurries. Net CO_2_ efflux was higher in topsoil slurries, which contained more dissolved organic carbon (DOC). CO_2_ efflux correlated well with DOC contents, suggesting the critical role of DOC. Microbial mechanisms contributed 9%–45% to CO_2_ production, as estimated by γ-ray sterilization experiments in the short-term reoxidation process. Solid-aqueous separation experiments showed a significant reduction in net CO_2_ efflux across all soil slurries after the removal of the original aqueous phase, indicating that the water phase was the main source of CO_2_ emission (>50%). Besides, C emission was greatly affected by pH fluctuation in acidic soil but not in neutral/alkaline soils. Fourier transform ion cyclotron resonance mass spectrometry and excitation-emission matrix results indicated that recalcitrant and macromolecular dissolved organic matter (DOM) components were more easily removed or attacked by •OH. The decrease in DOM content during the short-term reoxidation was the combined result of •OH oxidation, co-precipitation, and soil organic matter release. This study emphasizes the significance of the generally overlooked nonmicrobial mechanisms in promoting CO_2_ emission in the global C cycle, and the critical influence of the aqueous phase on C loss in paddy environments.

## Highlights

● CO_2_ efflux pulse event explained by nonmicrobial mechanisms mediated by O_2_.
● •OH promoted CO_2_ emission in paddy soil slurries during short-term oxidation.
● Aqueous phase was the main sources of CO_2_ emission during short-term oxidation process.
● For the DOM, easily biodegradable favors retention after the short-term oxidation.

## Graphical abstract

**Figure:**
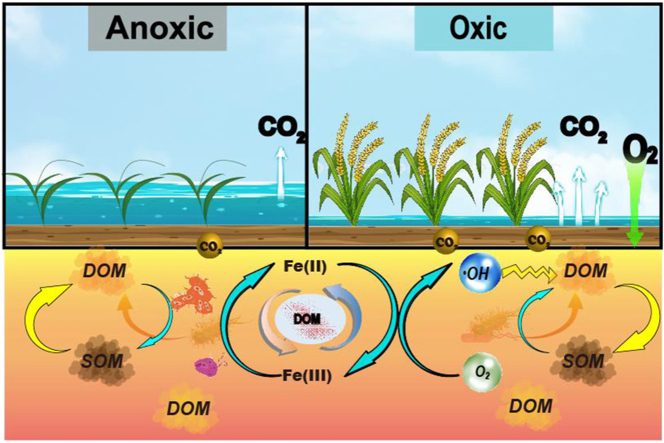

## 1 Introduction

The global emission of CO_2_ from soil is about seven times more than anthropogenic emissions [1]. Paddy soil is one of the most important constructed wetland soils, which has a higher organic carbon (C) storage than upland soils, with annual CO_2_ emissions beyond 1,309 g CO_2_/(m^2^⋅yr) [2,3]. Understanding the dynamic changes of paddy soil organic C is the key to mitigating global warming. In natural environments, the drying-wetting process occurs frequently in paddy soils, and previous studies have shown a sudden pulse event of CO_2_ efflux occurring after drainage [4]. Traditionally, the CO_2_ emission pulse occurs after exogenous labile C input, such as simple sugars, amino acids, biochar, etc. [[5], [6], [7], [8], [9]], which is called the “priming effect” [10]. The increasing CO_2_ concentration in the presence of exogenous C is mainly caused by enhanced microbial activities. Recently, it has been reported that nonmicrobial processes (e.g., Fenton oxidation and photodegradation) may strongly contribute to soil organic matter (SOM) decomposition [11]. However, the research on abiotic mechanisms of CO_2_ efflux pulse event is still in its infancy [12,13], and the mechanism has not been well defined, especially for CO_2_ emission during the flooding and draining alternating periods in paddy soils.

In natural environments (e.g., wetlands, paddy soil slurries, and sediments), periodic O_2_ limitation occurs. Hydroxyl radical (•OH) would be produced when the anaerobic environment is exposed to O_2_, which can mediate the mineralization of organic matter (OM) due to its high oxidative activity (E^0^ = 2.7 V) [14,15]. For example, the research by Tong et al. [16] suggested that •OH produced from the oxygenation of subsurface sediments could oxidize organic C and contribute to CO_2_ emission. A previous study proved that the abiotic pathway played an indispensable role in CO_2_ emission in acid sulfate soil after oxygenation [17]. In addition, Du et al. [18] demonstrated that •OH produced by Fenton reactions was an important oxidant for OM mineralization at residue/soil interfaces. Specifically, active Fe phases could regulate •OH formation and organic C transformation during drying-wetting cycles of paddy soil [19]. Although there have been several studies on the non-microbial processes (such as •OH oxidation) of OM mineralization, the contribution of abiotically-mediated CO_2_ emission at different depths and how paddy soil traits (e.g., OM content and types) affect abiotically-mediated CO_2_ emission have not been fully explored.

OM component is an important factor that determines the net CO_2_ emission. Dissolved organic matter (DOM) is the most labile fraction of SOM, which is very sensitive to the external environment, and thus even small changes could affect the emission of CO_2_ [20,21]. Although soil microorganisms are thought to be the main driver of DOM transformation, the abiotic processes involved cannot be neglected. Recent studies indicate that prolonged anoxia could promote the reductive dissolution of Fe-oxides, leading to an increase in dissolved organic carbon (DOC) pools by releasing OM from the iron complex [22]. After exposure to O_2_, the Fe^2+^ forms iron hydroxide precipitates, which could remove most of the DOM through coagulation (C protection by Fe complexation) [23]. The abiotic oxidation of Fe(II) is often accompanied by the oxidation of DOM to produce CO_2_ due to reactive oxygen species (ROS) production [24]. Therefore, the function of Fe in OC sequestration may be a double-edged sword. Under redox fluctuation conditions, Fe-mediated organic matter decomposition may counteract the protection effect [14], rendering changes in DOM quantity and composition unpredictable. Currently, research on the fate of DOM affected by environmental redox fluctuations and its relationship with CO_2_ emission is limited in actual soil conditions.

Here, we assessed the contribution of hydroxyl radical versus microbial processes to CO_2_ emission in different paddy soils, and the effect of these processes on the stability and chemical composition of DOM. The principal objectives of this study were to investigate: (1) the abiotically mediated CO_2_ emission pulse and •OH generation in different paddy soils or soils with different depths, (2) the contribution of nonmicrobial mechanisms to CO_2_ emission, and (3) changes in the chemo-diversity of DOM during the short-term reoxidation process. Our results underscore the role of abiotic mechanisms (e.g., •OH) in promoting organic matter mineralization, which may also have implications for efforts to enhance soil C stocks.

## 2 Materials and methods

### 2.1 The chemicals and paddy soils

Paddy soils at the depth of 0–20 cm (topsoil) and 40–60 cm (deep-soil) were collected from three different paddy fields in China, including Chengdu in Sichuan province (CD_0–20_/CD_40–60_, 104°14′E, 30°38′N), Yingtan in Jiangxi province (YT_0–20_/YT_40–60_, 116°54′E, 28°11′N), and Yangzhou in Jiangsu province (YZ_0–20_/YZ_40–60_, 119°28′E, 32°19′N). The chemicals and the physicochemical properties of paddy soils used in this study are supplied in Support information (SI) (Text S1 and Table S1).

### 2.2 Anaerobic incubation and reoxidation experiments

Paddy soil slurries were prepared by mixing air-dried paddy soils (passing through a 100-mesh sieve) with sterile ultrapure water at a ratio of 1:2.5 in 60 mL serum bottles. Then, all samples are degassed with nitrogen (N_2_, 99.99%) to remove oxygen (O_2_) and re-establish anoxic conditions. The bottles were sealed with butyl rubber septa, crimped with aluminum caps, and shaken in the dark (at 180 rpm and 25 °C). During 20 d of anoxic incubation, the concentrations of dissolved Fe(II) and total Fe (II/III) in paddy soil slurries were determined by the UV–Vis spectrophotometer method [25] (See *Section* 2.4.1). Similar to our previous study [26], Fe(II) concentration reached a relatively stability level after 18 d of incubation (Fig. S1a). The pH and Eh of slurries were monitored with a portable meter (HACH, HQ30D, USA) during the incubation period (Fig. S1b). All the experiments were tested in triplicate and carried out in the anoxic glovebox (Braun Co, Germany), and all chemical reagents were prepared with degassed water. After the anoxic incubation, all headspace of samples were purged, and filled with 99.99% N_2_ (to facilitate the determination of CO_2_ generated during the reoxidation process). To ensure pressure balance, 10 mL headspace gas was extracted before the same volume of O_2_ (about 450 μmol) was injected into the headspace of bottles to initiate the oxidation reactions. The anoxic controls used the same volume of N_2_ instead of O_2_ to determine the effect of reoxidation. Then, these bottles were shaken at 180 rpm in the dark for 4 h (at 25 °C). At specific time intervals, 20 mL of headspace gas was withdrawn for measuring headspace CO_2_ concentration with gas chromatography (Agilent 7890A, USA). All the oxygenation experiments were conducted in triplicate, and destructive sampling was employed. The CO_2_ efflux rate can be described as [6]:

$$R=\frac{C\times V\times M}{22.4\times m\times t}$$

where *R* is the CO_2_ efflux rate [μg C/(g dry soil⋅min)]; *C* is the measured CO_2_ concentration (ppm); *V* is the effective volume of the incubation jar (30 mL); *M* is the molar mass of C (12 g/mol); 22.4 L is the molar volume of an ideal gas at 1 atm; *m* is the gram dry weight of the soil (g); *t* is the time (min).

To evaluate the contribution of solid and aqueous phases to CO_2_ emission, paddy soil slurries were centrifuged at 5,000 rcf for 30 min after 20 d of anoxic incubation. After removing the aqueous phase, the solid phase was mixed with the same volume of degassed ultrapure water, which was performed in the anoxic glove box.

### 2.3 Detection of ROS during the reoxidation of different paddy soils

#### 2.3.1 Chemical probe methods

The cumulative concentration of •OH during the reoxidation of paddy soils was measured using benzoic acid (BA) as a probe, which can be transformed to *p*-hydroxybenzoic acid (*p*-HBA) by •OH [19,27], and details are presented in Text S2. To gain a fuller understanding of the reaction mechanism during the reoxidation period, the production of O_2_•^−^ and H_2_O_2_ was also measured using the 2,3-bis-(2-methoxy-4-nitro-5-sulfophenyl)–2H-tetrazolium-5-carboxanilide (XTT, 0.05 mM) method and the 10-acetyl-3,7-dihydroxyphenoxazine (ADHP) method [28,29], respectively. Details are provided in Text S2.

#### 2.3.2 Electron paramagnetic resonance (EPR) characterization

To further verify the generation of ROS during the reoxidation process, EPR methods were applied to detect O_2_•^−^ and •OH production. 5,5-dimethylpyrroline N-oxide (DMPO) was applied to semi-quantitatively analyze the level of •OH and O_2_•^−^ in paddy soil. DMPO solutions (0.3 mL, 0.1 M) in water or methanol were mixed with different anoxic samples (0.2 mL) and analyzed with Bruker EMXmicro-6/1/P/L spectrometer (Karlsruhe, Germany). The operating parameters for EPR analysis are provided in Text S3.

#### 2.3.3 Quenching experiments

To test the contribution of abiotic (e.g., •OH) and biotic processes to C transformation in paddy soil redox conditions, quenching experiments were used to evaluate the role of the produced •OH in soil respiration. Here, KI was used as •OH scavenger. Briefly, 1.0 mL of freshly prepared KI solution was added to the anoxic bottles, and the I^−^ concentration reached 1 mmol/g dry soil [30]. γ-Irradiation at 50k Gray was used to inhibit microbial and enzyme activities in soil slurries to represent the abiotic processes. Sterilization was confirmed using the plate counting method (Fig. S2), and previous studies showed that •OH formation was not affected by γ-irradiation [19].

### 2.4 Chemical analyses and characterization

#### 2.4.1 Sequential extraction of Fe(II) species from paddy soil

Sequential extraction of Fe(II) species in the paddy soil was carried out according to the procedures described in the previous study [19]. Fe(II) was measured at 510 nm with a microplate reader (Tecan Infinite 200 Pro, Switzerland) using the 1,10-phenanthroline method. Details of extraction steps are shown in Text S4.

#### 2.4.2 Characterizations of organic C

Paddy soil DOM was extracted at the end of oxic or anoxic incubation. The DOC content, spectral characteristics, and compositional information of soil DOM were analyzed using TOC analyzer, 3-D excitation-emission matrix (EEM), and Fourier transform ion cyclotron resonance mass spectroscopy (FT-ICR-MS), respectively. Details can be found in Text S5.

## 3 Results and discussion

### 3.1 •OH production and O_2_-mediated CO_2_ release of soil slurries

**Fig. 1:**
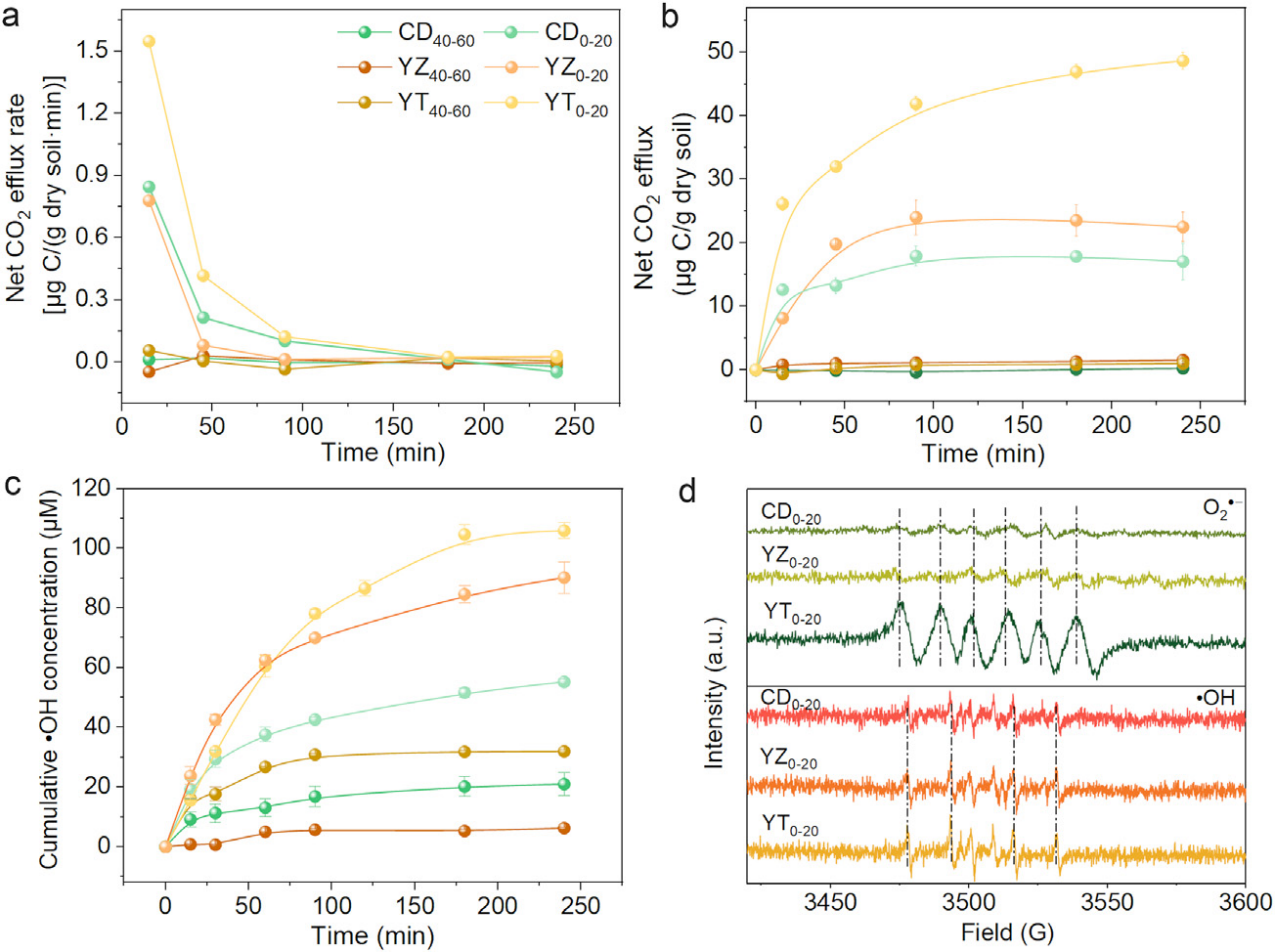
Net CO_2_ efflux rates (a) and cumulative net emission of CO_2_ (b) over the reoxidation period of 4 h. Cumulative concentration of •OH in different paddy soil slurries (c), and EPR spectra of •OH and O_2_•^−^ in different paddy soils under aerobic conditions (d). YT_0–20/40–60_, CD_0–20/40–60_, and YZ_0–20/40–60_ represent the paddy soil in Yingtan (0–20 cm/40–60 cm), Chendu (0–20 cm/40–60 cm), and Yangzhou (0–20 cm/40–60 cm), respectively.

The physicochemical properties of paddy soils are presented in Table S1. During the 20 d of anoxic incubation, Eh decreased at first and then leveled off, indicating that the soil microcosms reached a stable state. After O_2_ input, C mineralization was measured (Fig. S3). The results showed that the CO_2_ efflux rate was high in the initial period and then began to decrease, possibly due to O_2_ depletion. In the three kinds of topsoil slurries, higher cumulative CO_2_ was observed in the O_2_ atmosphere (52.7–115.5 μg C/g dry soil) than in the control (30.5–60.3 μg C/g dry soil). Throughout the reoxidation period, the net C mineralization rate was higher in topsoil [0.02–1.5 μg C/(g dry soil⋅min)] than in deep-soil slurries [0–0.05 μg C/(g dry soil⋅min)]. For different soils, the mineralization rate followed the order of YT_0–20_ > YZ_0–20_ > CD_0–20_ in the early stages (i.e., the first 45 min) (Fig. 1a). After 4 h of reoxidation, the net cumulative CO_2_ emissions of YT_0–20_ (48.68 μg/g dry soil), CD_0–20_ (22.5 μg/g dry soil), and YZ_0–20_ (17.1 μg/g dry soil) were much higher than those of YT_40-60_ (1.5 μg/g soil), CD_40–60_ (0.8 μg/g dry soil), and YZ_40-60_ (1.7 μg/g dry soil) (Fig. 1b). These results indicated that O_2_ input significantly promoted the emission of CO_2_ in the three paddy soils with different depths, resulting in positive priming. Compared with topsoil slurries, deep-soil slurries released a lower amount of CO_2_.

The production of •OH in soil slurries was then measured. Compared to the N_2_ atmosphere (Fig. S4d), O_2_ input extensively promoted the generation of •OH in all samples (Fig. 1c). The highest cumulative •OH was detected in YT_0–20_ (106.0 μM), followed by YZ_0–20_ (90.2 μM), CD_0–20_ (55.3 μM), YT_40-60_ (32.0 μM), CD_40–60_ (21.0 μM) and YZ_40-60_ (10.4 μM), which agreed with the trend in net C mineralization rates. EPR technique was used to detect the formation of •OH after feeding O_2_. An EPR signal with an intensity of 1:2:2:1 was observed (Fig. 1d), indicating that •OH was produced in the three different soils. Previously, Huang et al. [31] measured the concentration of •OH on the surface of soil minerals with EPR technique, and found that •OH mineralized organic matter into CO_2_. Trusiak et al. [32] also found that CO_2_ production under aerobic conditions was due to •OH formation.

**Fig. 2:**
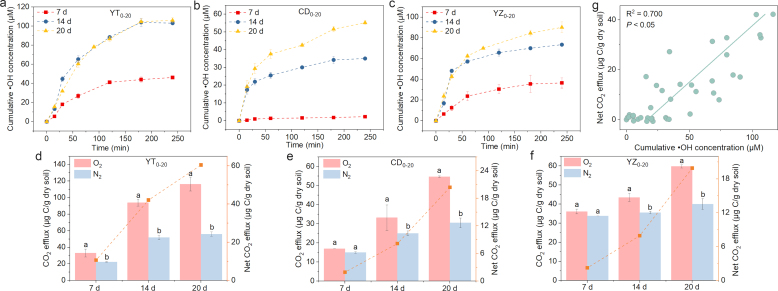
The cumulative concentration of •OH over time (a–c) and CO_2_ efflux under O_2_ or N_2_ conditions in topsoil slurries with incubation time of 7, 14 and 20 d (d–f). Correlation of net CO_2_ efflux with cumulative •OH concentration (g). Different lowercase letters in the same group indicate significant difference (*P* < 0.05) among different treatments for the same soil.

Increasing the concentration of electron acceptors (O_2_) could produce more ROS (e.g., •OH) (Figs. S4a–c), which may promote the emission of CO_2_. On the other hand, since the redox state affects •OH formation and subsequent CO_2_ emission after O_2_ incorporation, the influence of anaerobic incubation time was examined, by conducting oxygenation experiments after 7, 14, and 20 d of incubation. The results showed that the cumulative concentration of •OH and CO_2_ emission gradually increased with increasing anaerobic incubation time in three topsoil slurries (Fig. 2a–f), suggesting that high redox state favored ROS formation and C mineralization. This is due to the fact that •OH generation during redox fluctuation depends on Fe(II) (the main electron-donating contributor), while more Fe(III) can be reduced to Fe(II) by microorganisms as time proceeds. The •OH accumulation exhibited significant linear relationships to CO_2_ emission in the topsoil slurries (R^2^ = 0.629, *P* < 0.05) (Fig. 2g).

O_2_•^−^ and H_2_O_2_, the key intermediates for •OH production, might be generated during soil slurry oxygenation. Subsequently, the generation of O_2_•^−^ and H_2_O_2_ was monitored during the reoxidation period. As shown in Fig. S5, the cumulative O_2_•^−^concentrations were 7˗11 times higher in topsoil samples than in deep-soil samples after 4 h of oxidation. The spin-trapping EPR results also indicated that O_2_•^−^ was formed in the topsoil slurries (Fig. 1d). Previous research has shown that coastal soil would work as an electron-storing geobattery material, triggering one-electron transfer and the generation of O_2_•^−^ and H_2_O_2_ during high and low tides [33]. Similarly, the paddy soil slurries acted as reduced geobattery materials during the alternating flood and drought, and transferred one electron to O_2_ to produce O_2_•^−^, which further transformed into H_2_O_2_ via dismutation by the Haber–Weiss mechanism [34]. As expected, limited H_2_O_2_ was produced in N_2_ conditions (Fig. S6).

### 3.2 Biotic and abiotic contribution to CO_2_ emission

**Fig. 3:**
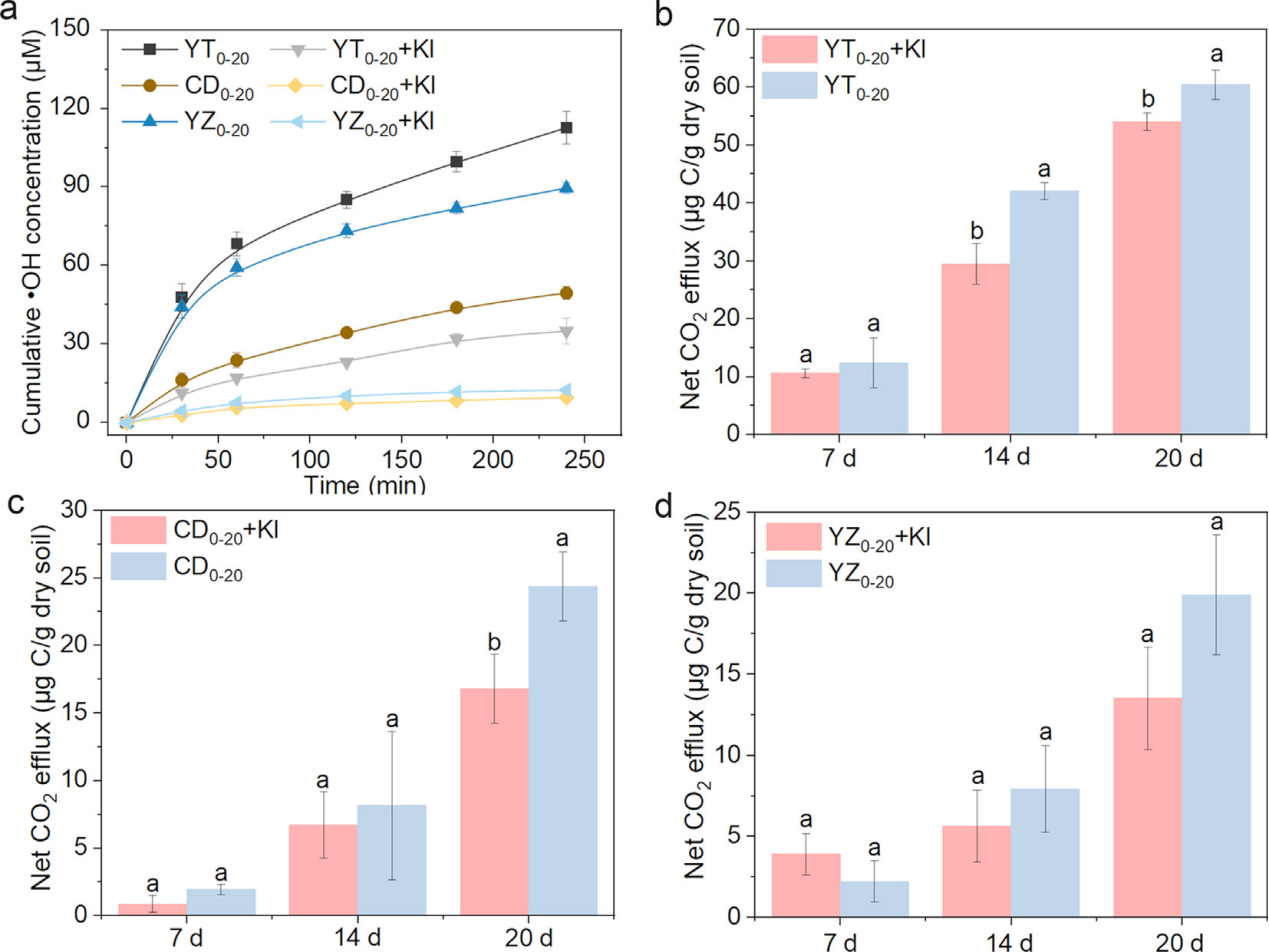
The generation of •OH in the presence or absence of •OH quencher (KI) within 4 h (a), and change of net CO_2_ emission in the presence of KI in topsoil slurries with anoxic incubation time of 7, 14 and 20 d (b–d).

Soil CO_2_ can be produced by both biotic and abiotic processes [35,36], and the latter might contribute to CO_2_ release to a greater extent in a relatively short period after O_2_ perturbation. To investigate the effects of •OH on CO_2_ emissions, KI (1 mmol/g dry soil) was added to the soil slurries as •OH quencher before feeding O_2_. Importantly, to prevent the mineralization of exogenous C by •OH, alcohols (organic compounds) were not selected as quenchers of •OH. Adding KI eliminated about 69%–84% of •OH (Figs. 3a, S7a), and a previous study has demonstrated that such concentration of I^−^ has little influence on soil microorganisms after 4 h of reaction [30]. The addition of KI decreased 14.8%–30% of net CO_2_ emission of topsoil samples during 4 h oxidation (Figs. S7b and S8). Surprisingly, quenching of •OH in deep-soils reduced 40%–77% of net CO_2_ emission, which was mainly due to the low metabolism of microorganisms in deep-soils (Fig. S7c and d). Likewise, the abiotic decomposition of organic matter in desert soils with very low SOM content was comparable to the mineralization process carried out by living organisms [37]. A previous study has reported that exposure of pure lignin-derived organic matter (OM) system to •OH originating from Fenton-type reactions generates condensed aromatic and alicyclic aliphatic compounds [38], which was likely part of the processes involving ring opening, polymerization, and/or cyclization and hydrogen abstraction. Additionally, the •OH-mediated oxidation of OC could alter its molecular structure and chemical composition via hydroxylation or the cleavage of aromatic rings to finally produce low-molecular-weight compounds or CO_2_ [39]. The produced low-molecular-weight compounds would be more accessible to microbial utilization. On the other hand, the Fenton reaction combined with the main oxidative enzymes produced significant amounts of CO_2_, and the CO_2_ efflux with lignin peroxidase was 10-fold that of abiotic Fenton reaction without enzymes [40].

We also evaluated the contribution of •OH to CO_2_ generation in soils after different anoxic incubation periods (Fig. 3b–d). The contribution of •OH was negligible after a short period of anoxic incubation (7 d); after 20 d of incubation, net CO_2_ efflux showed varying degrees of reduction in KI treatments in comparison to that of unamended soils, indicating the specific role of •OH in CO_2_ pulse. Considering the complexity of the soil environment, the reaction of DOM with •OH can generate DOM• (a highly reactive state), which can continue to react with H_2_O_2_ or soil enzymes, etc., to produce CO_2_ [41]. Processes responsible for CO_2_ generation in oxic paddy soil slurries may be related to enzymes. A recent study suggested that the Fenton reaction combined with the main oxidative enzymes produced significant amounts of CO_2_, and the CO_2_ efflux with lignin peroxidase was 10-fold that of abiotic Fenton reaction without enzymes [40]. Combined with the quench experiment, the results demonstrated that the increased CO_2_ production was most likely derived from the production of ROS, and our results underscore the importance of •OH in promoting SOM mineralization. Typically, DOM is the most active component of SOM, which might be attacked by ROS to generate CO_2_. The changes in DOC will be discussed later.

Moreover, other abiotic processes, such as carbonate dissolution caused by pH changes, could also affect CO_2_ production. To evaluate the contribution of carbonate, slurry pH was measured after reoxidation. After 4 h of oxidation, the pH of the acid soil (YT_0–20_) decreased by 1.02 ± 0.3 unit, while the pH of the neutral soils (CD_0–20_ and YZ_0–20_) only decreased by 0.20 ± 0.1 and 0.22 ± 0.05 units, respectively, indicating a smaller release of inorganic carbon in neutral soils (Fig. S9a). To further evaluate the contribution of pH decline to CO_2_ emission, 0.1 M HCl was introduced to the slurry under anaerobic conditions, effectively reducing the pH of YT_0–20_ by 1.0 units. After acidification, CO_2_ concentration was measured. We observed a doubling of CO_2_ emission immediately, with a net emission of 30.5 μg C/g dry soil (Fig. S9b). Remarkably, this increase in CO_2_ emission accounted for about 50% of the total net emissions from YT_0–20_. These findings underscore the crucial role of pH in regulating CO_2_ emissions during short-term oxidation in acid soil systems.

Based on the results of γ-ray sterilization, microorganisms also played a role in C emission during the short-term oxidation process. The microorganisms in YT_0–20_ exhibited low activity in response to O_2_ perturbation, contributing only 8% to C emission, while those in CD_0–20_ and YZ_0–20_ contributed 43% and 40%, respectively (Fig. S10). It has been reported that when anoxic conditions are changed to oxic, C mineralization is significantly accelerated after 48 h, compared to permanently anoxic conditions [42]. Although microbial respiration may dominate in the long run, overall, our results proved that non-microbial processes (such as ROS oxidation or carbonate) played critical roles in the CO_2_ pulse emission during short-term oxidation.

### 3.3 Relative contributions of solid and aqueous phase in CO_2_ emission

DOM plays an important role in energy source and electron transfer during the alternation of oxic and anoxic processes [43]. Conversely, the proportion of recalcitrant C represents the chemical recalcitrance of SOM [9]. In this study, the net CO_2_ emission was negatively correlated with the proportions of recalcitrant C (*P* < 0.05) (Fig. S11), suggesting that the labile C pool was the main source of CO_2_ efflux.

To elucidate the contribution of solid and aqueous phases of soil slurries in •OH formation and CO_2_ emission, solid-aqueous separation experiments were performed. The cumulative concentration of •OH in the solid phase (55–106 μM) was comparable to that in the mixed slurries, while low levels of •OH (6–16 μM) were detected in the aqueous phase (referring to the original aqueous phase, OAP) (Fig. S12), indicating that •OH was mainly derived from the solid phase. The total Fe(II) oxidized by O_2_ or ROS in the solid phase ranged from 12.9 to 14.4 mM, and the electron utilization efficiency for •OH production in the solid phase followed the order of CD_0–20_ < YZ_0–20_ < YT_0–20_ (Table S2). The aqueous phases generated a lower amount of •OH, but exhibited much higher electron utilization efficiency (0.26%–0.64%) than the solid phases (0.13%–0.26%) (Table S2). High electron utilization efficiency may be related to the C/Fe ratio or DOM types [29,44]. Solution pH was the key factor affecting ion-exchangeable Fe(II) oxidation, and previous studies indicated that Fe(CO_3_)_2_^2−^ or Fe^2+^/FeOH^+^ were the dominant species to describe Fe(II) oxidation at a pH higher or lower than 6.0, respectively [45]. The oxidation rate of Fe(CO_3_)_2_^2−^ was approximately three orders of magnitude higher than that of FeOH^+^ [26], indicating that higher pH favored the oxidation of ion-exchangeable Fe(II). Overall, the results indicated that the aqueous phases contributed little to •OH formation when exposed to O_2,_ and the formed •OH showed a high correlation with Fe(II) species.

**Fig. 4:**
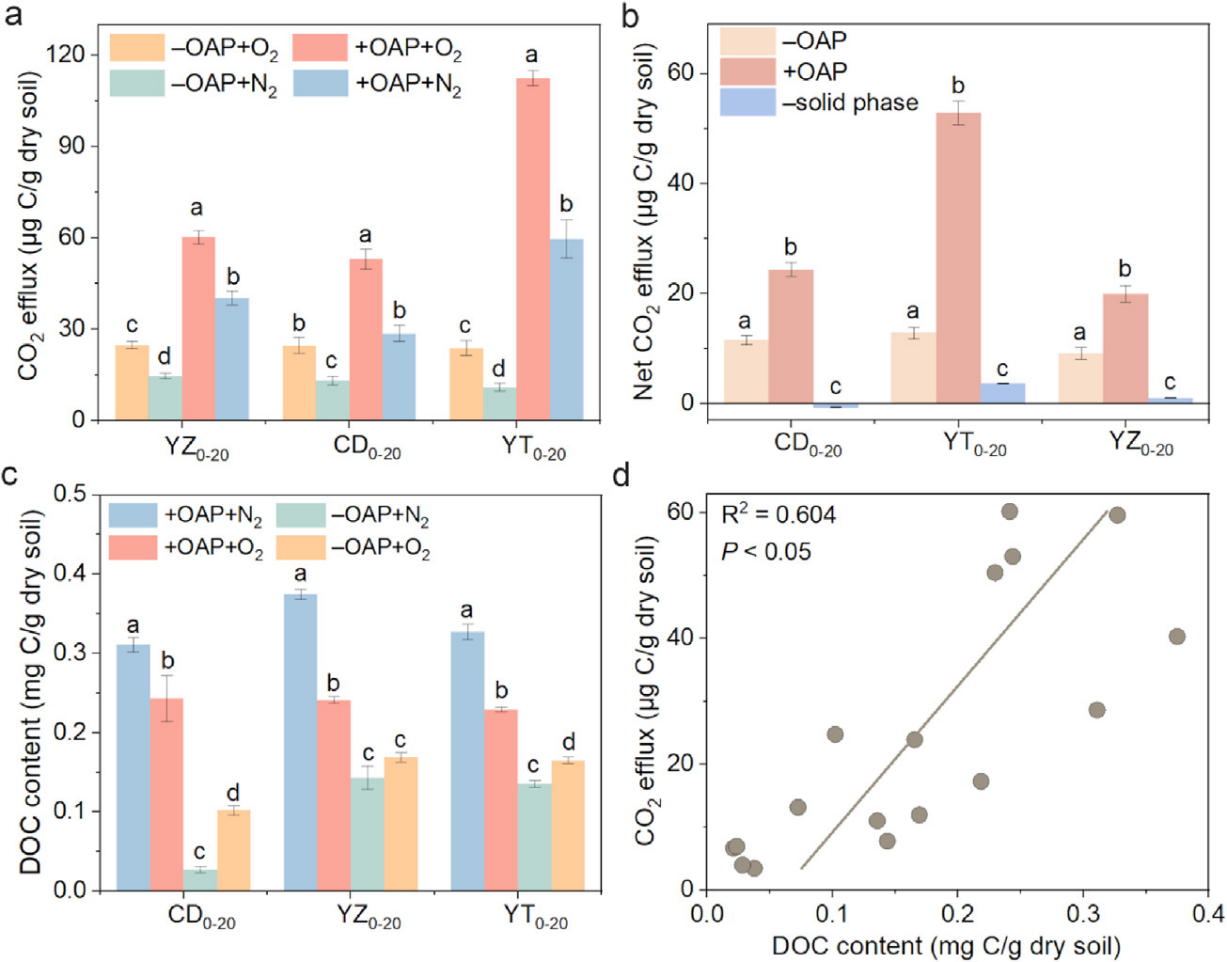
CO_2_ efflux with or without OAP (a) and net CO_2_ efflux in different topsoil slurries (b) in O_2_/N_2_ atmosphere after 4 h of reaction. DOC content of soil slurries with and without OAP in O_2_/N_2_ atmosphere after 4 h of reaction (c). Correlation analyses between CO_2_ efflux and the content of DOC (d). +OAP, with original aqueous phase; –OAP, without original aqueous phase; –solid phase, only aqueous phase.

In the OAP, O_2_ input did not cause the CO_2_ emission pulse (Fig. S13a). This was possible because •OH formation was low in the aqueous phase, resulting in minimal mineralization of DOM. Furthermore, the concentration of Fe^2+^ was also at low levels (Table S2), and introducing O_2_ into the solution had a limited impact on pH levels and did not disrupt the carbonate balance (to release CO_2_). After the removal of OAP, the CO_2_ emission of three solid phases were inhibited in varying degree in the N_2_ atmosphere (Fig. 4a), suggesting that dissolved CO_2_ was mainly in the OAP. Further, O_2_ perturbation increased CO_2_ emissions of solid phases, regardless of whether there was OAP, and a higher net CO_2_ emission (by 110%–309%) was observed in the presence of OAP (Fig. 4a and b). Notably, the net CO_2_ emissions of YT_0–20_ exhibited the most significant decrease after the removal of OAP, highlighting its high sensitivity to pH change. However, the reduction in net CO_2_ emissions cannot be solely attributed to the inorganic carbonate removal in the aqueous phase, and the aqueous phase also contains a significant amount of DOC. Compared to the solid phase, the aqueous phase had more DOC and dissolution of carbonates, which was the primary source of CO_2_ emissions (>50%), leading to decreased emission of CO_2_ when OAP was removed. A recent study showed that DOC of topsoil in a subtropical forest was the major cause of rain-induced soil CO_2_ pulse [46]. Therefore, the short-term mineralization of OC was most likely derived from the aqueous phases, although •OH was mainly formed in the solid phases.

The effect of O_2_ addition on DOC content in soil slurries was also surveyed. In the presence of the OAP, the concertation of DOC of three different slurries decreased after O_2_ addition (Fig. 4c). The DOC loss was ascribed to Fe-organic matter coprecipitation and C mineralization by the abiotic (e.g., •OH) or biotic process, as supported by previous research, which showed that O_2_ perturbation would result in adsorption and coprecipitation of a large fraction of DOM with Fe(III), Al(III) or Si(IV) at anoxic-oxic water–soil interface [23,47]. Conversely, when the aqueous phases were removed (replaced by ultrapure water), DOC content increased after O_2_ addition (Fig. 4c), which may be due to the dissolution of SOM. On the other hand, it was reported that Fe(II) oxidation could stimulate ROS formation and microbial respiration, and then facilitate SOM decomposition [11,48,49]. Additionally, the OM biodegradability also increased with enhanced oxygen availability [49]. DOC content almost did not decrease in the OAP after O_2_ addition (Fig. S13b), being consistent with the trend of CO_2_ emission (Fig. S13a). Overall, CO_2_ emission was significantly correlated with DOC content (R^2^ = 0.604, *P* < 0.05) (Fig. 4d), suggesting that high DOC content benefits organic C mineralization in these paddy soils, which is controlled by biotic and abiotic processes.

### 3.4 Spectral characteristics and molecular composition of DOM

**Fig. 5:**
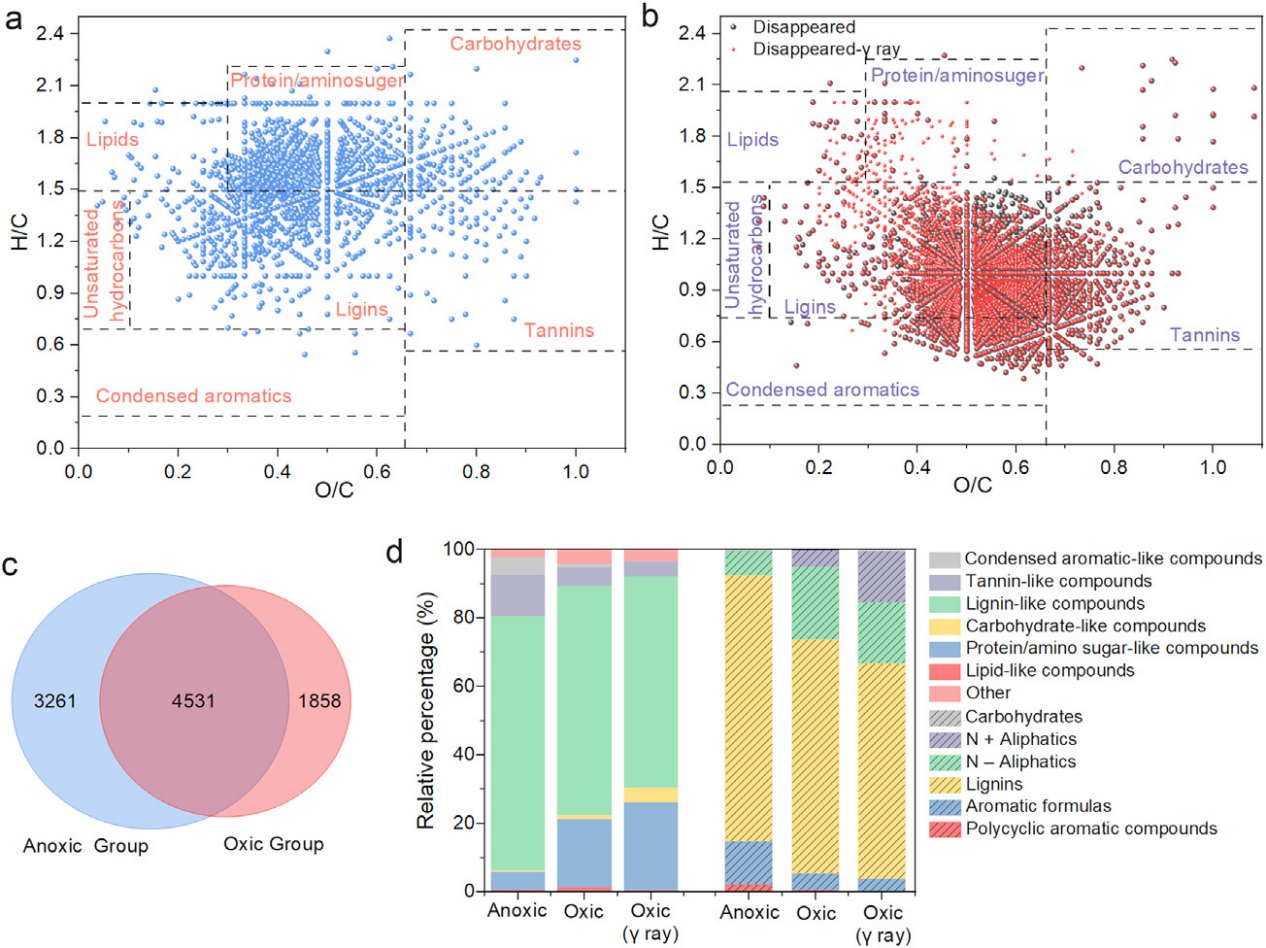
Van Krevelen diagrams from the Fourier transform ion cyclotron resonance mass spectrometry (FT-ICR-MS) spectra of YT_0–20_ slurry DOM. The produced (a) and disappeared (b) unique compounds formulas after oxygenation. Venn analysis of the molecular formulas from anoxic and oxic groups (c), and the proportion of different DOM components in anoxic and oxic groups (d).

FT-ICR-MS was used to identify DOM compositions by detecting unique molecules in the YT_0–20_ slurry (due to the high •OH yield and OC contents). A Venn plot (Fig. 5c) showed that the anoxic treatment group shared 4,531 compounds with the oxic treatment group from the overall 7,792 DOM molecules, and the van Krevelen diagram showed the removed formulas after oxygenation (Fig. 5b). Similar formula removal was observed in the γ-ray sterilization group after oxidation (sharing 80% of their total molecules), which indicated that the abiotic processes dominated the specific changes of DOM. The removed formulas mainly included lignin-like, tannin-like, and condensed aromatic-like compounds (Fig. S15i). The proportion of low molecular weight (MW) DOM increased, and a similar trend was observed for both aromatic index (AI) and double bond equivalence (DBE) (Fig. S15a–c), reflecting that the aromaticity and unsaturation degree decreased after oxidation, and that macromolecular DOM was more prone to be removed from the aqueous phase by oxidation or co-precipitation. •OH has the potential to initiate an attack (or addition) on the carbon atom of the benzene ring, resulting in ring-opening or hydroxylation [38], which may lead to a decrease in DBE. According to the definition by Lv et al. [50], the long-term storage of SOM in soils has a “Matthew effect”. Specifically, aromatic compounds with biochemical resistance in DOM have a strong affinity for soil minerals, while some compounds that are easy to biodegrade often have a low affinity for soil minerals. This study obtained similar results in that the aromaticity macromolecular DOM proportion decreased, and a higher proportion of protein/amino sugar-like compounds (easily biodegradable) were observed after the oxidation process (Fig. 5a, d). Moreover, carboxylic-rich alicyclic molecule (CRAM) compounds, as part of recalcitrant DOM, were detected at about 60% (by intensity) in all removed formulas (Fig. S15g). These results suggested that labile DOM was more easily utilized by microorganisms or mineralized by •OH during the oxidation process. Previous research has also indicated that Fe(II) oxidation increases organic C availability, which would also stimulate microbial respiration rates [49].

Under redox fluctuation conditions, changes in DOM are also influenced by soil pH. Therefore, the decrease in DOM content resulted from co-precipitation and SOC release, in addition to •OH attack. Venn plot demonstrated that anoxic and oxic treatments shared 2,377 molecules when OAP removal. That also meant that the oxic treatment group contained 90% of the molecules in the anoxic treatment group, and the anoxic treatment group also had fewer unique compounds than the oxic treatment group (Fig. S15h). The increased DOM mainly included CHON and CHOS, and CHO components (Fig. S16d–f).

Five different fluorescent components (C1, C2, C3, C4, and C5) were identified by the EEM-PARAFAC analysis (Fig. S17). C1 (Ex/Em: 250/450 nm), C3 [Ex/Em: 210(275)/400 nm] and C4 (Ex/Em: 225/450 nm) are generally related to UVC humic-like components, while C2 [Ex/Em: 225(275)/300 nm] and C5 [Ex/Em: 275(240)/350 nm] are known as protein-like substances (tyrosine-like and tryptophan-like, respectively). Similar peaks can be found in previous studies [51,52]. The total fluorescence intensities (F_max_) of five different fluorescent components decreased after oxidation, like the results of FT-ICR-MS. The fluorescence index (FI) values exceeded 1.8 in all samples, indicating that the DOM was derived from microbial sources. Strong linear relationships between FI and CO_2_ emission also suggested that DOC produced by microorganisms was the main source of C mineralization (Fig. S18b, *P* < 0.01). Similarly, the biological index (BIX), which is associated with bacterial activities, also demonstrated positive relationships with CO_2_ emission (Fig. S18a). Conversely, the humification index (HIX) value also exhibited a salient negative correlation with CO_2_ emission fluxes (*P* < 0.05) (Fig. S18d), suggesting that lower HIX favor OM decomposition and CO_2_ emission. O_2_ perturbation reduced HIX in different treatments (Fig. S18c), indicating that the degree of humification and the amount of high-MW DOM were decreased after O_2_ addition, consistent with the FT-ICR-MS results. Li et al. [53] reported similar results that the HIX decreased along with increased oxygen availability. In all treatments, the O_2_ addition led to more microbial-derived substances (higher FI), lower humification degree (lower HIX), and smaller molecular weight (lower S_R_, Fig. S18f).

## 4 Conclusion

Soil carbon emission or storage is closely related to soil fertility and global climate change. C emission mediated by abiotic factors has been largely underestimated during the short-term oxidation of paddy soils. Our results demonstrated that the nonmicrobial mechanisms (ROS oxidation and carbonate dissolution) played an important role in CO_2_ emission during the short-term oxidation, while only 9%–45% of carbon emission originated from microbial processes. The main conclusions of this study were as follows: (i) Correlation analyses and quench experiments showed that •OH mediated about 10%–77% of CO_2_ release (directly or indirectly) from OM, and the contribution of •OH oxidation to CO_2_ production in deep-soil slurries were higher than that in topsoil slurries; (ii) Intensity of abiotically mediated CO_2_ efflux pulse was greater in soils with longer anaerobic incubation time; (iii) Although •OH was mainly formed in the solid phase, CO_2_ emission was largely derived from the DOC of the aqueous phase; (iv) For the DOM, recalcitrant compounds favored escape while easily biodegradable retention after the short-term oxidation. Findings from this work provide new insights into the nonmicrobial mechanisms of CO_2_ release. Further studies are required to gain a deeper understanding of radical mechanisms in real rice field ecosystems.

## Author contributions

J.S.L.: conceptualization, data analysis, original draft preparation, and data curation. C.Y.Z.: investigation, and review & editing. X.T.L.: Methodology. X.L.W.: Investigation. D.M.Z.: funding acquisition, supervision, and resources.

## Declaration of competing interests

The authors declare no conflicts of interest.
