# Supplementary material for: Nonmicrobial mechanisms dominate the release of CO2 and the decomposition of organic matter during the short-term redox process in paddy soil slurry
Source: Eco Environ Health. 2023 Aug 29;2(4):227–34. doi: 10.1016/j.eehl.2023.08.005 (PMC10902510; doi:10.1016/j.eehl.2023.08.005)
Supplement: Multimedia component 1 [file mmc1.docx]

**Supporting Information**

**Nonmicrobial mechanisms dominate the release of CO_2_ and** **the decomposition of organic matter during the short-term redox process in paddy soil slurry**

Jinsong Liu ^1^, Changyin Zhu ^1, *^, Xiantang Liu ^1^, Xiaolei Wang ^1^, Dongmei Zhou ^1, *^

*^1^ State Key Laboratory of Pollution Control and Resource Reuse, School of the Environment, Nanjing University, Nanjing 210023, China*

^*^*Corresponding authors.*

E-mail address: [cyzhu@nju.edu.cn](mailto:cyzhu@nju.edu.cn) (C.Y. Zhu)

[dmzhou@nju.edu.cn](mailto:dmzhou@nju.edu.cn) (D.M. Zhou).

Number of pages: 26

Number of Texts: 5

Number of Tables: 3

Number of Figures: 18

**Texts**

**Text S1. Materials.** Calcium chloride (CaCl_2_, 98%), hydrogen peroxide (H_2_O_2_, 30%), benzoic acid (BA, 99%), 1,10-phenanthroline (98%), sodium acetate (98%) *p*-hydroxybenzoic acid (*p*-HBA, 99%) were purchased from J&K Scientific Ltd, China. 5.5-Dimethyl-1-pyrroline-N-oxide (DMPO, 97%) was purchased from DOJINDO, Japan. Hydroxyamine hydrochloride (98%), hydrochloric acid (GR), sulfuric acid (GR), Dimethyl sulfoxide (DMSO, 99%), and phosphoric acid (HPLC grade, 85%) were obtained from the National Medicines Corporation Ltd. (Beijing, China). Methanol (HPLC grade) was obtained from TEDIA Company (USA). All solutions were prepared using ultrapure water from a Synergy UV ultrapure water system with a Millipak-40 filter unit (Millipore Corporation Merck KGaA, Darmstadt, Ge).

**Text S2.** **ROS Detection.** The experiments were conducted according to the procedure described in our previous studies [1]. Briefly, 100 mM BA solution (2 mL, degassed with N_2_) was added into the anaerobic bottles before oxidation, and the anoxic controls were also performed in the N_2_ atmosphere. Then, 1.0 mL of different soil slurries were withdrawn using a graduated syringe (without opening caps) and transferred into 5 mL centrifuge tubes with 0.5 mL methanol immediately at various preselected time intervals during oxidation process. The mixture was centrifuged for 1 min at 9000 rcf, and filtered through 0.22 μm nylon membrane syringe filter. The concentrations of *p*-HBA in filtrate were measured using high-performance liquid chromatography (HPLC, Shimadzu, Japan) equipped with a 25 cm × 4.6 mm Cosmosil C18 column. The mobile phase was a mixture of 0.1% phosphoric acid solution and acetonitrile (40:60, v/v) at a flow rate of 0.5 mL min^-1^ with the detection wavelength of 254 nm. The detection limit was 0.1 µM for *p*-HBA. Cumulative •OH concentrations were estimated by the 5.87 times of *p*-HBA concentrations (C_•OH_ = 5.87 × C*_p_*_-HBA_).

2,3-bis-(2-methoxy-4-nitro-5-sulfophenyl)–2H-tetrazolium-5-carboxanilide XTT) formazan is a product of XTT with O_2_^•-^. The concentration of this product was detected by UV–Visible spectrophotometer (UV–vis, Thermo Scientific, USA) at 475 nm. The concentration of O_2_^•-^ was calculated by using the equation: $\text{C=(A×v)/(ε×L×2)}$. C is the concentration of generated O_2_^•-^, A is the absorbance of XTT formazan, v is the volume of total sample (20 mL), ɛ is the extinction coefficient of XTT formazan (23800 M^-1^cm^-1^), L is optical path length (1 cm), and 2 is the conversion factor of O_2_^•-^mediated reaction with XTT. Cumulative H_2_O_2_ concentration was quantified by using the Ampliflu Red (ADHP) method. It can react with H_2_O_2_ in the presence of horseradish peroxidase (HRP). The concentrations of HRP and ADHP were 20 mg/L and 2 mM, respectively. The filtered sample was diluted and transferred into the 96-well plates (less than 30 s). The fluorescence of the sample was measured by a microplate reader (Tecan Infinite 200 Pro, Switzerland) (excitation wavelength = 558 nm, emission wavelength = 588 nm), and control calibration was tested using a background solution.

**Text S3. Electron paramagnetic resonance (EPR) parameters.** The extracts were injected into special quartz capillaries and then inserted into the cavity of the EPR spectrometer (Bruker EMXmicro-6/1/P/L, Germany). The operating parameters were given below: modulation frequency of 4.0 G, center field of 3500 G, modulation amplitude of 4.0 G, sweep width of 200 G, microwave frequency of 9.8 GHz, resolution of 1400 points, and sweep time of 10 s.

**Text S4. Fe sequential extraction.** 1 M CaCl_2_ (pH 7.0) was used for extracting ion-exchangeable Fe, 0.5 M HCl for the surface-adsorbed Fe and Fe in low-crystalline minerals, and 5 M HCl for Fe in high-crystalline minerals, respectively. For CaCl_2_ extractable Fe and SOC, 1 mL soil slurry (~0.31 g dry soil) was extracted by 5 mL 1M CaCl_2_ at 200 rpm for 2 h, followed by centrifugation (1 min at 9000 rcf) and filtration of the supernatant through 0.22 μm syringe filters for Fe(II) measurements. The filtrate was preserved with 1 M HCl in the glove box until analyses. Then, the remaining soil pellets were re-suspended in 5 mL 0.5 M HCl and shaken for 2 h at 200 rpm, then centrifuged, and filtered (1 min at 9000 rcf). After that, the remaining soil pellets were re-suspended in 5 mL 5 M HCl and shaken for 24 h, then centrifuged, and filtered (1 min at 9000 rcf). All the experiments were tested in triplicate and carried out in the anoxic glovebox. Fe(II) was measured at 510 nm with a microplate reader (Tecan Infinite 200 Pro, Switzerland) using the phenanthroline method [2].

**Text S5.** **Dissolved** **organic matter (DOM)** **analysis**. The supernatant was centrifuged and filtered through a 0.45 μm filter, and the concentration of DOC was measured by a Vario Select TOC analyzer (vario TOC, Germany). The absorbance of DOM samples (diluted 10 times) was scanned with a UV−Vis spectrophotometer (TU-1810, Beijing, China) from 200-600 nm with 0.5 nm increments. To further confirm the transformation of DOM compositions during re-oxidization periods, 3-D excitation-emission matrix (EEM) fluorescence spectra of slurry DOM samples were measured with an F-7000 fluorescence spectrophotometer (Hitachi, Japan). The concentration of all samples was diluted below to 5 mg C L^−1^ with Milli-Q water before measurements. The excitation wavelength (Ex) (200-500 nm) and emission wavelengths (Em) (250-550 nm) were scanned with 5 nm intervals. Blank samples from Milli-Q water were measured before actual samples. Moreover, the correction of Rayleigh scattering peaks, Raman normalization, as well as parallel factor (PARAFAC) analysis modeling, was achieved with MATLAB 2022a. Spectral slope ratios (S_R_, defined as S_275–295_/S_350–400_), which indicate the molecular mass of DOM. Humification index (HIX, Peak area under the emission wavelengths at 435–480 nm divided by the peak area at 300–345 nm, with excitation at 254 nm), fluorescence index (FI, the ratio of emission intensities at 470 nm and 520 nm, with excitation at 370 nm), and biological index (BIX) were calculated based on excitation-emission matrix spectroscopy (EEM).

The content of recalcitrant SOM was determined using the two-step acid hydrolysis method [3, 4]. Basically, paddy soil samples were first hydrolyzed with 2.5 M H_2_SO_4_ at 105 °C for 30 min. The hydrolysates were centrifuged and decanted. The residue was washed with distilled water and the supernatant was added to the hydrolysate. The remaining residue was further hydrolyzed with 13 M H_2_SO_4_ and shaken overnight at room temperature. Subsequently, the distilled water was added to dilute the acid concentration to 1 M, and the sample was hydrolyzed at 105 °C for 3 h. The remaining soil residue was rinsed twice with distilled water and dried at 60 °C. This fraction was considered the recalcitrant SOM pool. The relative abundances of the recalcitrant C were calculated as the ratio of the SOC content of different soil samples to the total SOC.

Furthermore, Fourier transform ion cyclotron resonance mass spectroscopy (FT-ICR MS, Bruker SolariX, 15.0 T) was used for distinguishing the molecular composition of DOM, and the data was calculated with TRFu batch code on MATLAB [5]. Solid phase extraction (SPE) was proceeded by using Varian Bond Elute PPL cartridges (Agilent, 500 mg, 6 mL) to remove the salt from all collected samples. PPL cartridges were wetted with 50 mL of 100% HPLC-grade methanol, followed by acidified Milli-Q water (50 mL, pH < 2), and then 20 mL samples were passed through the cartridges by gravity at a flow rate of approximately 2 mL min^-1^. Cartridges were rinsed with 18 mL of 0.01 M HCl for removal of salts, dried with a stream of N_2_ and immediately extracted with 6 mL of methanol (MS grade). These dried samples were dissolved in 1 mL of 50:50 methanol/water (v/v) for ultrahigh resolution mass spectrometry analysis. Additionally, standard Suwannee River Natural Organic Matter solution (50 mg L^-1^) (obtained from IHSS) was used for calibration. The samples were characterized using negative-ion mode electrospray ionization with a 12 T superconducting magnet Bruker Daltonics Apex Qe FT-ICR-MS instrument. Ammonium hydroxide was added immediately prior to negative mode electrospray to increase the ionization efficiency. Samples were injected by a syringe pump providing an infusion rate of 120 μL h^-1^ and analyzed with the electrospray voltages optimized for each sample to maintain consistent and stable ion currents. Spectra were performed over 400 scans in the range of 200-900 m/z with an ion accumulation time of 0.6 s, giving about a 38 min total run time for each sample. The spectra of SPE blank and solvent blank were also analyzed at the same time to correct the obtained profiles for DOM samples.

| **Name** | **pH** | **OM**  **(g kg^-1^)** | **OC**  **(g kg^-1^)** | **CEC**  **(cmol kg^-1^)** | **Fe (mg kg^-1^)** | **Mn (mg kg^-1^)** | **Soil particle content of different grain grades (g kg^-1^)** | | | **Soil texture**  **(Made in USA)** |
| --- | --- | --- | --- | --- | --- | --- | --- | --- | --- | --- |
|  |  |  |  |  |  |  | **Clay**  **(~0.002mm)** | **Powder grain**  **(0.002~0.05mm)** | **Sand**  **(0.05~2.0mm)** |  |
| CD_0-20_ | 6.69 | 18.4 | 10.7 | 11.3 | 22271 | 328 | 218 | 544 | 238 | Silt (sand) loam |
| CD_40-60_ | 7.22 | 2.55 | 1.5 | 5.90 | 14380 | 140 | 117 | 512 | 371 | Silt (sand) loam |
| YZ_0-20_ | 7.26 | 36.1 | 20.9 | 19.8 | 28606 | 564 | 239 | 646 | 115 | Silt (sand) loam |
| YZ_40-60_ | 8.11 | 7.06 | 4.1 | 13.4 | 35237 | 640 | 251 | 657 | 92 | Silt (sand) loam |
| YT_0-20_ | 4.85 | 24.5 | 14.2 | 13.6 | 33296 | 110 | 335 | 345 | 320 | Clay loam |
| YT_40-60_ | 5.54 | 5.70 | 3.3 | 10.06 | 32457 | 103 | 273 | 243 | 484 | Sandy clay loam |

Table S1. Physicochemical properties of paddy soil samples.

Table S2. Electrons released by Fe(II) and electron utilization efficiencies for •OH production ^a^.

| **Name** | **Cumulative** **•OH (μM)** | **C_0 Fe(II)_**  **(mM)** | **C_t Fe(II)_**  **(mM)** | **∆Fe(II) (mM)** | **Transformation efficiency for •OH (%)** | **Electron utilization efficiency for •OH (%)** |
| --- | --- | --- | --- | --- | --- | --- |
| CD_0-20_  (Solid, +O_2_) | 55.25 | 37.76 | 23.73 | 14.04 | n.a. | 0.13 |
| YZ_0-20_  (Solid, +O_2_) | 90.26 | 43.56 | 30.67 | 12.90 | n.a. | 0.23 |
| YT_0-20_  (Solid, +O_2_) | 106.00 | 45.54 | 31.80 | 13.74 | n.a. | 0.26 |
| CD_0-20_  (Liquid, +O_2_) | 11.90 | 0.74 | 0.02 | 0.62 | n.a. | 0.64 |
| YZ_0-20_  (Liquid, +O_2_) | 5.98 | 0.76 | 0.01 | 0.70 | n.a. | 0.29 |
| YT_0-20_  (Liquid, +O_2_) | 16.32 | 1.81 | 0.05 | 1.51 | n.a. | 0.40 |
| CD_0-20_  (+H_2_O_2_) | 49.70 | 32.94 | 12.69 | 20.24 | 4.97 | 0.082 |
| YZ_0-20_  (+H_2_O_2_) | 50.90 | 44.35 | 15.75 | 28.60 | 5.09 | 0.059 |
| YT_0-20_  (+H_2_O_2_) | 55.49 | 50.58 | 10.74 | 32.85 | 5.55 | 0.046 |

^a^ For the sake of simplicity, only the data results at the final time points were used for estimation.

n.a. = data not available.


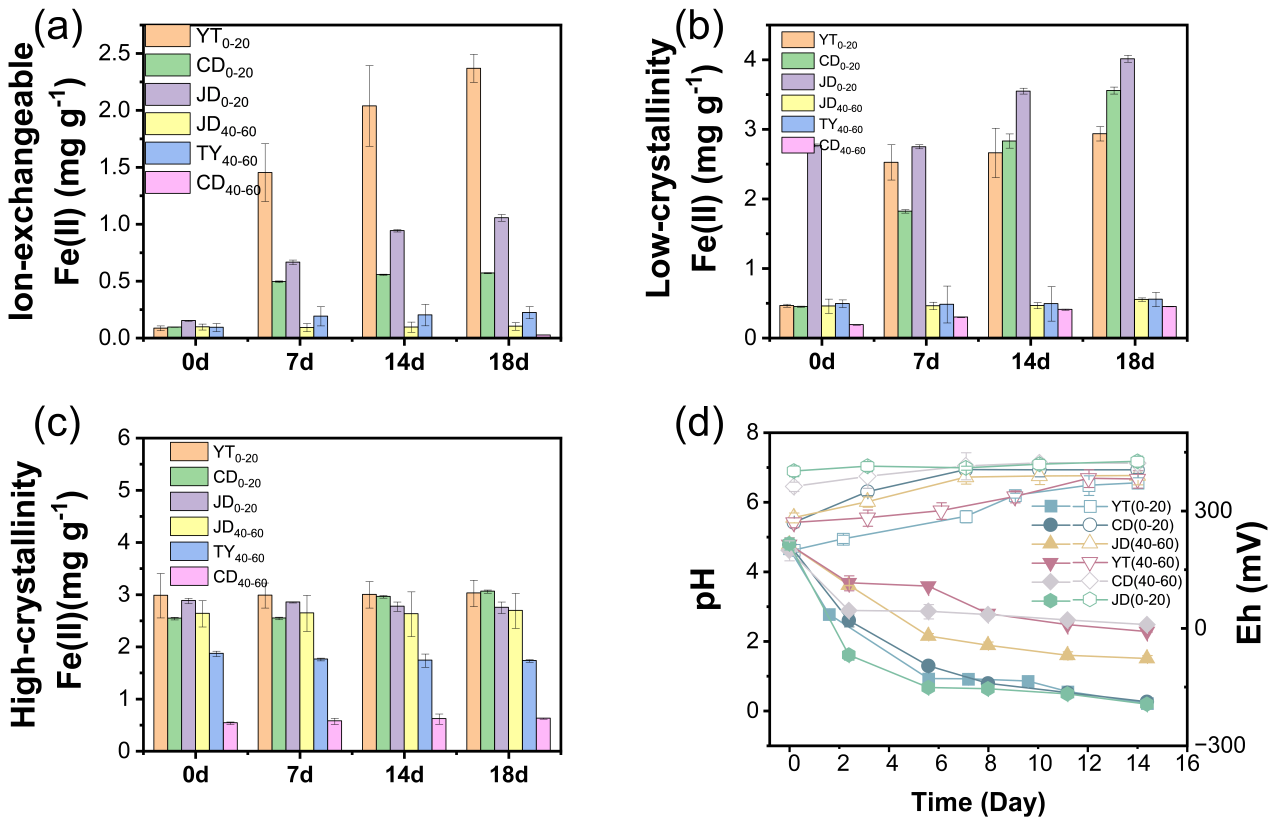


Figure S1. Changes of (a) ion-exchangeable Fe(II), (b) low-crystallinity Fe(II), and (c) high-crystallinity Fe(II). (d) Eh and pH in the six kinds of paddy soil slurries during anoxic incubation (Eh: solid; pH: hollow).


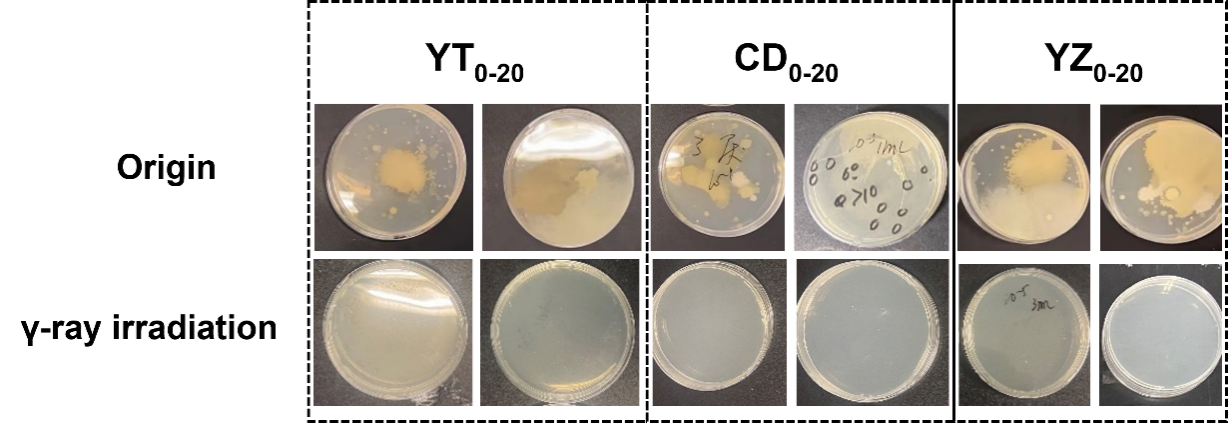


Figure S2. Plate culture images of topsoil slurries before and after irradiation.


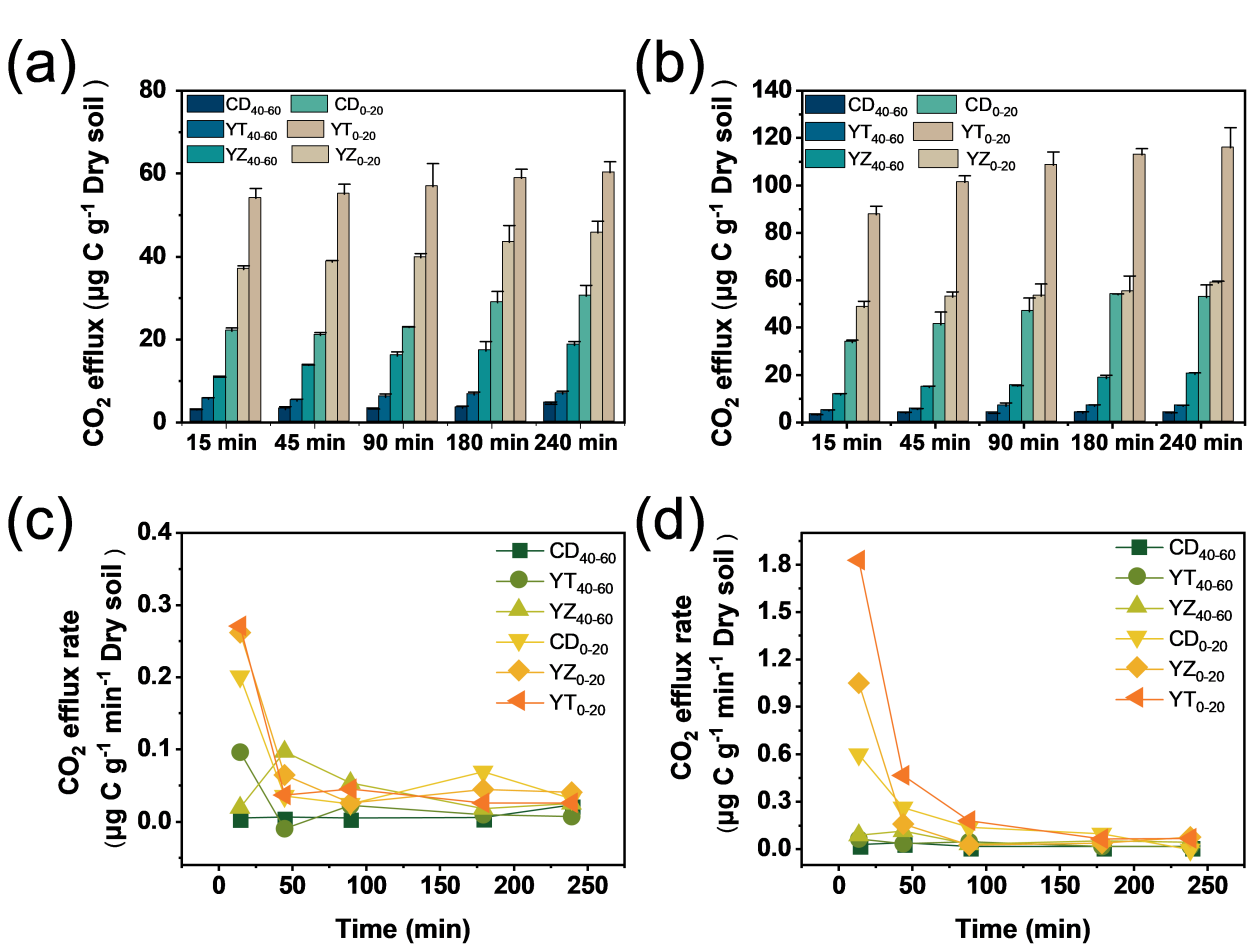


Figure S3. Accumulated concentration of CO_2_ of different paddy soil slurries in (a) N_2_ atmosphere and (b) O_2_ atmosphere. CO_2_ efflux rate of different paddy soil slurries in (c) N_2_ atmosphere and (d) O_2_ atmosphere.


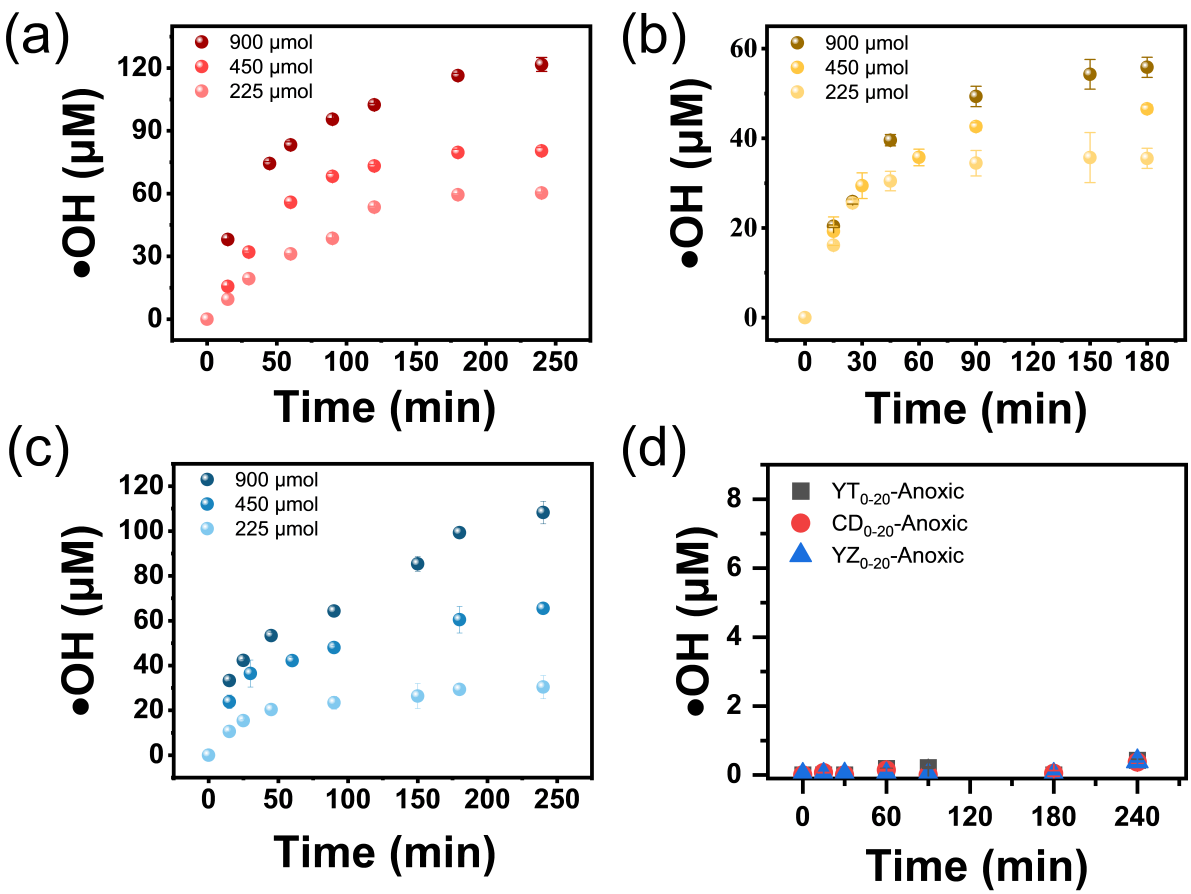


Figure S4. Effect of O_2_ concentration on •OH accumulation in (a) YT_0-20_, (b) CD_0-20_, (c) YZ_0-20_ slurries. (d) •OH accumulation in CD_0-20_, YZ_0-20_ and YT_0-20_ slurries under anoxic (N_2_ atmosphere) conditions.


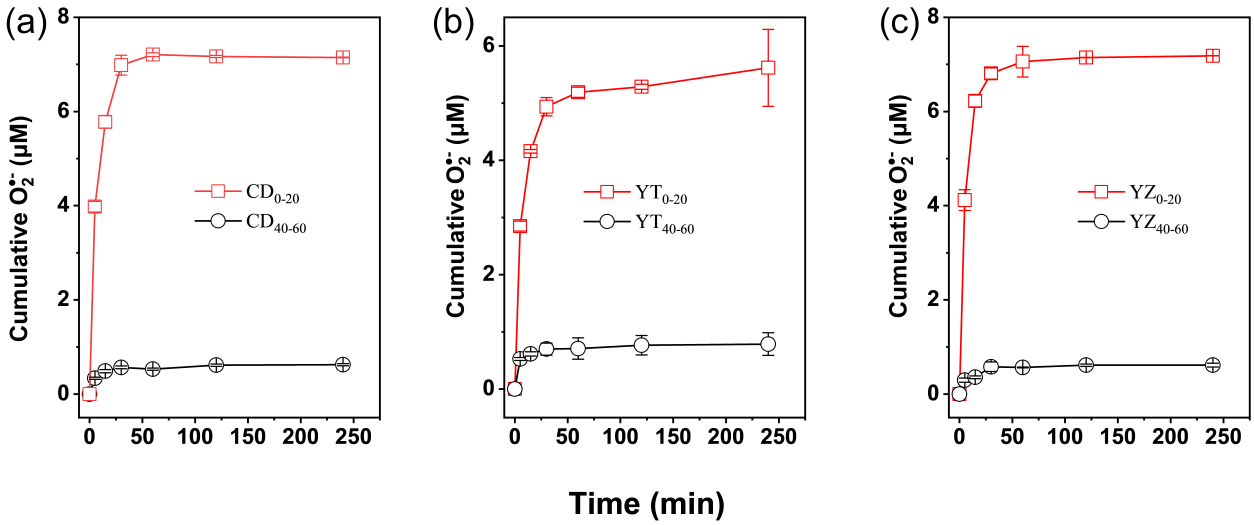


Figure S5. Time series of cumulative O_2_^•−^ production in (a) CD_0-20/40-60_, (b) YT_0-20/40-60_, (c) YZ_0-20/40-60_ under O_2_ conditions.


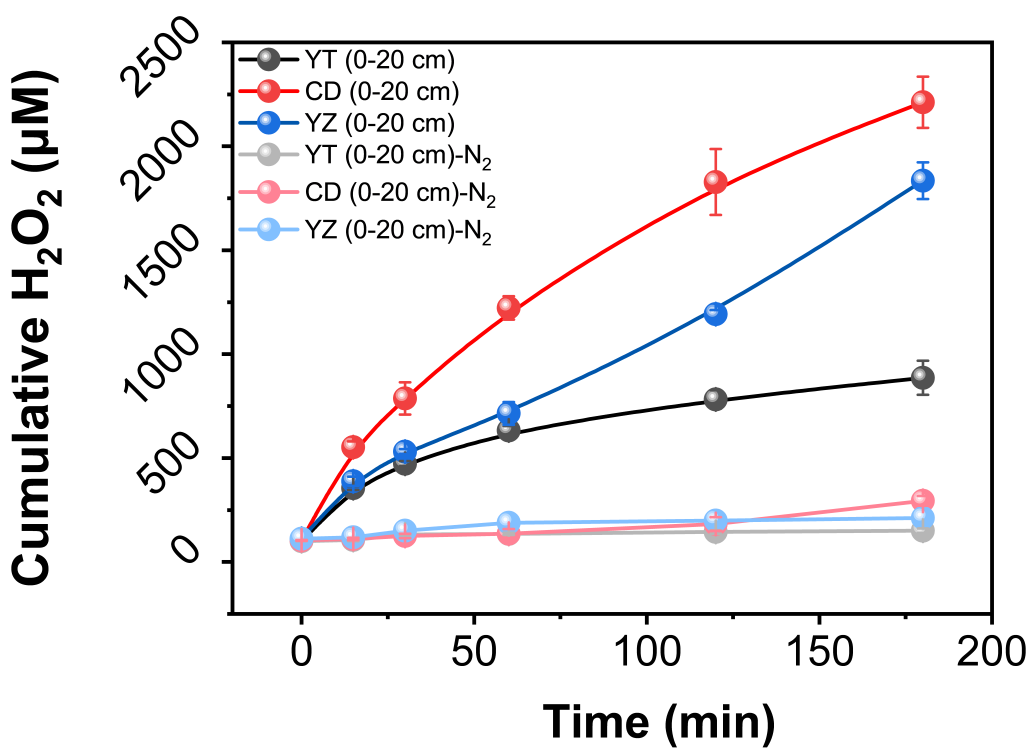


Figure S6. Cumulative H_2_O_2_ concentration of three kinds of topsoil samples in N_2_ or O_2_ atmospheres.


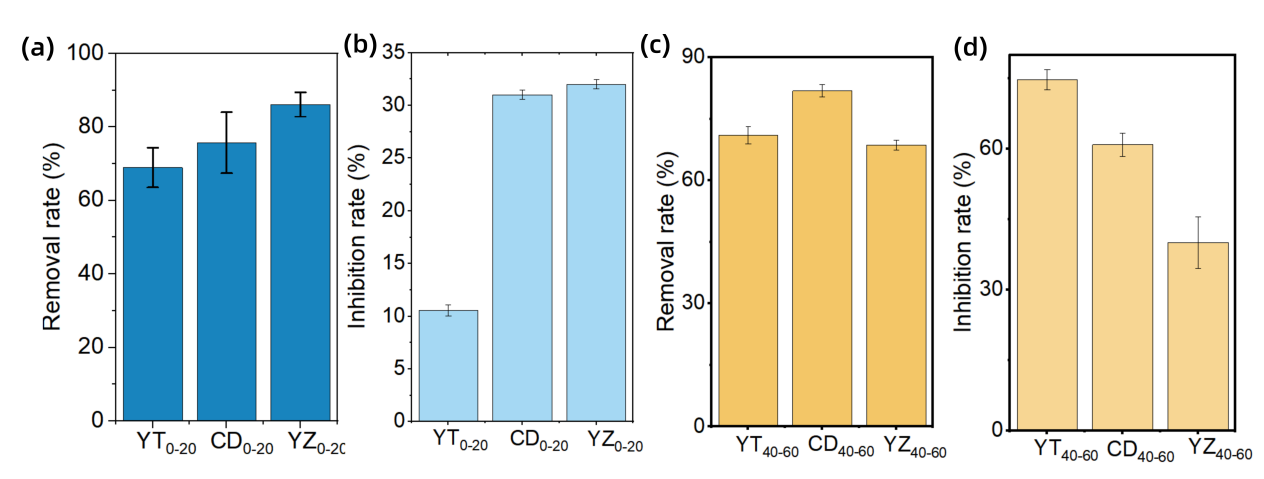


Figure S7. The quenching efficiency of •OH in (a) topsoil and (c) deep-soil slurries. Effects of KI on net CO_2_ efflux in (b) topsoil and (d) deep-soil slurries.


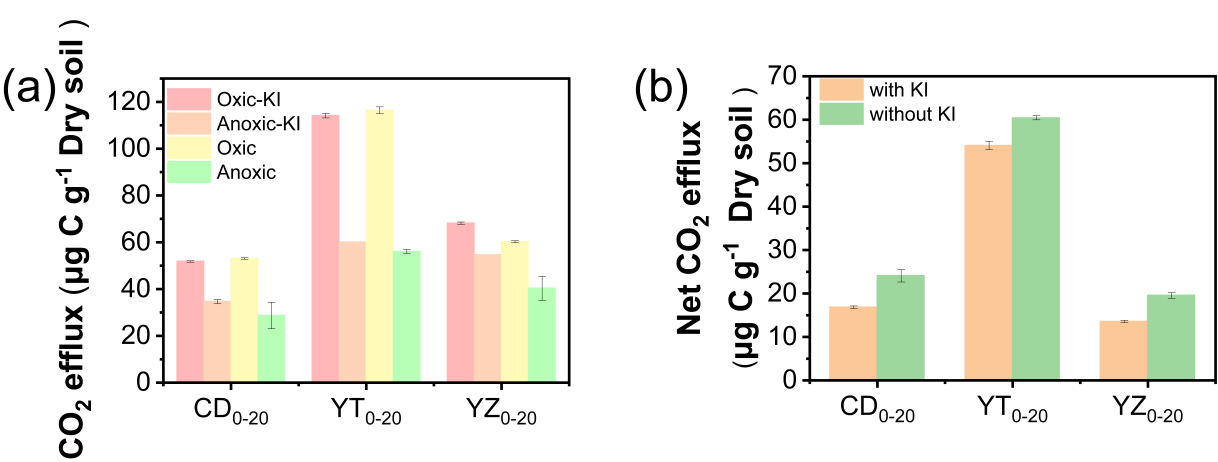


Figure S8. (a) CO_2_ efflux and (b) net CO_2_ efflux in unamended / KI addition treatment groups in oxic / anoxic conditions.


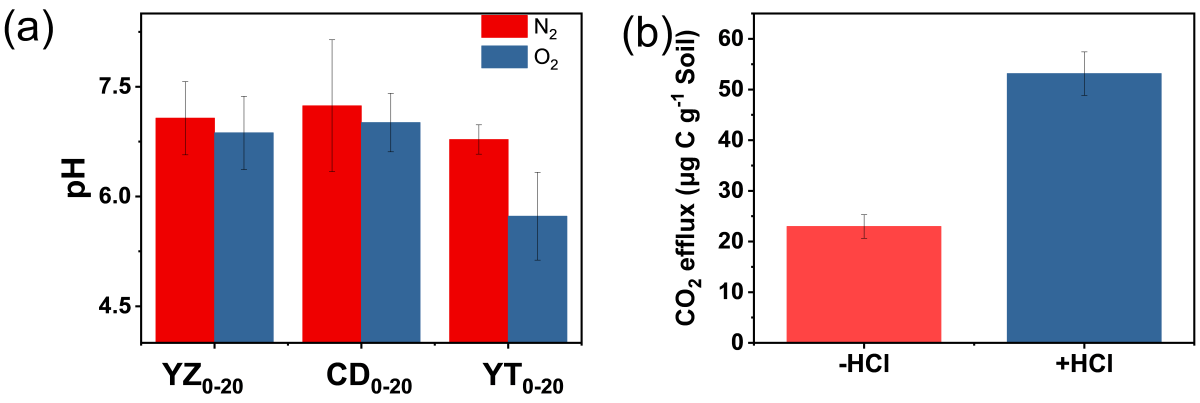


Figure S9. (a) Changes of pH of the slurries after the oxygenation, (b) production of CO_2_ from HCl-amended soil slurry (YT_0-20_).


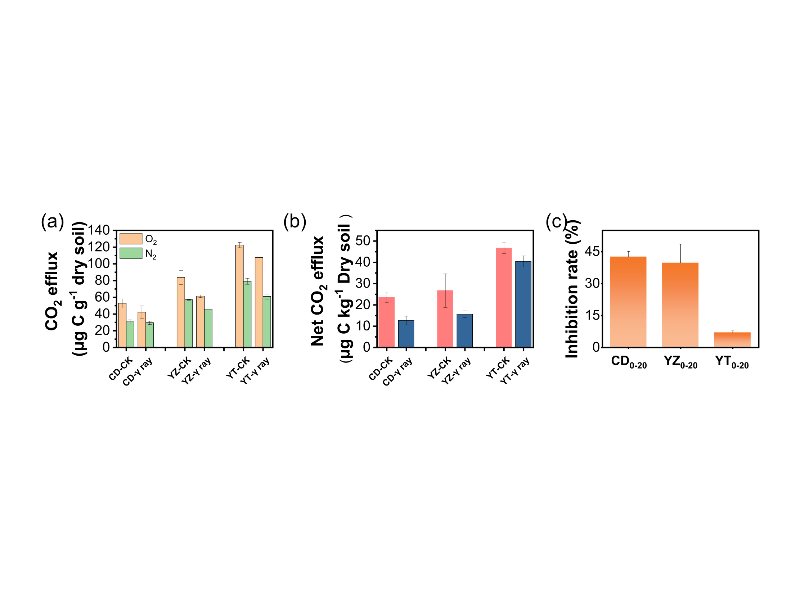


Figure S10. (a) CO_2_ efflux and (b) net CO_2_ efflux in unamended / sterilization treatment groups; and (c) the contribution of microorganisms on CO_2_ emission in three slurries.


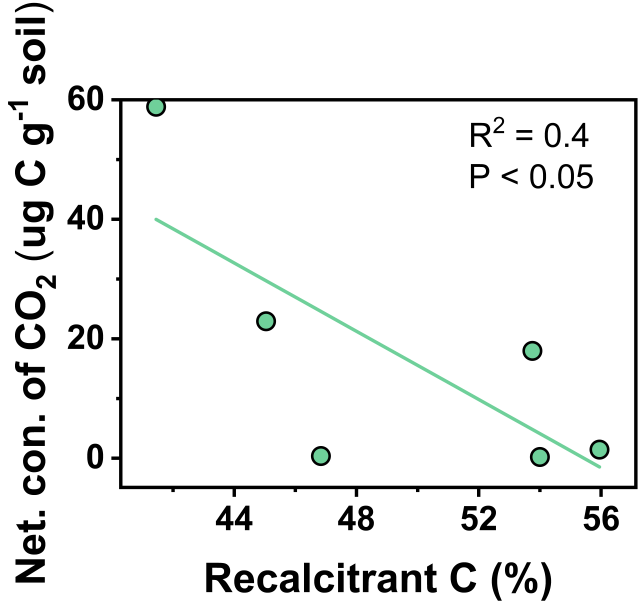


Figure S111. Relationships between the net CO_2_ efflux and the recalcitrant C after oxidation reaction.


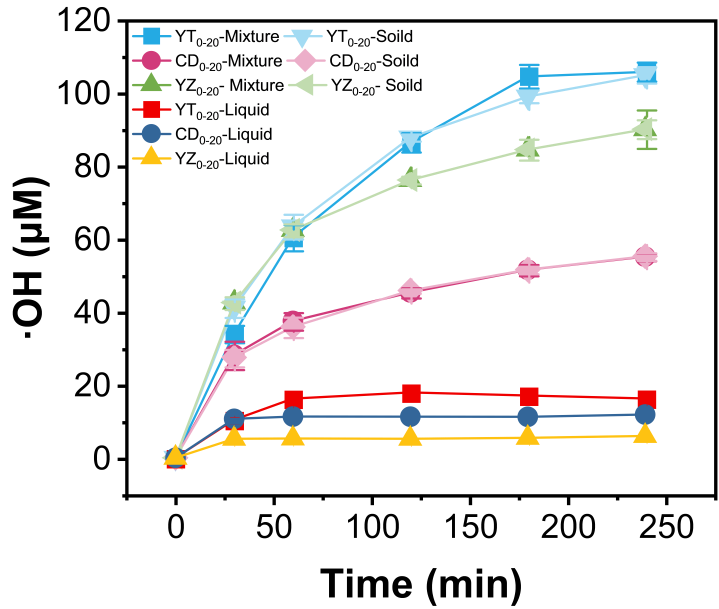


Figure S12. •OH production of solid and aqueous phases of topsoil slurries after oxygenation.


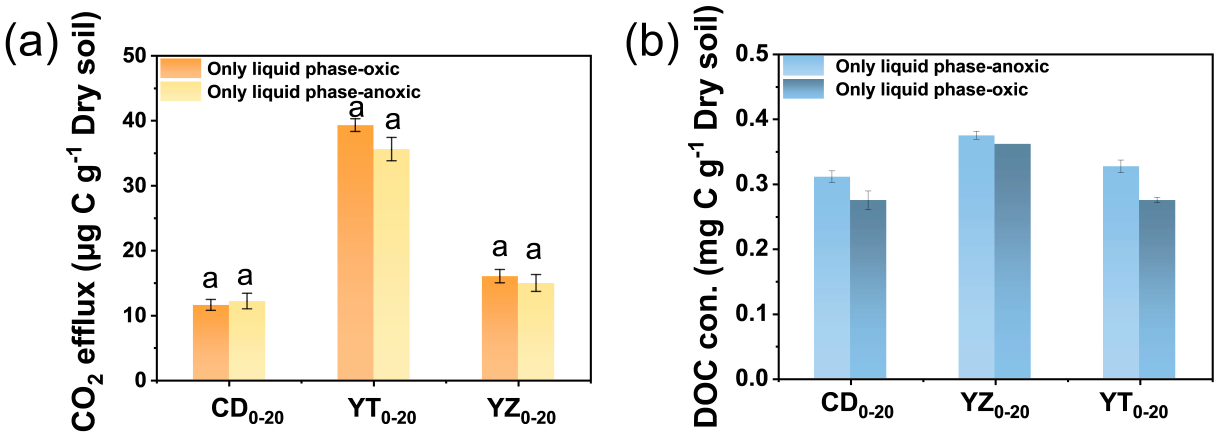


Figure S13. (a) CO_2_ efflux and (b) DOC concentration of the liquid phase (without filtration), in O_2_/N_2_ atmosphere after 4 h reaction.


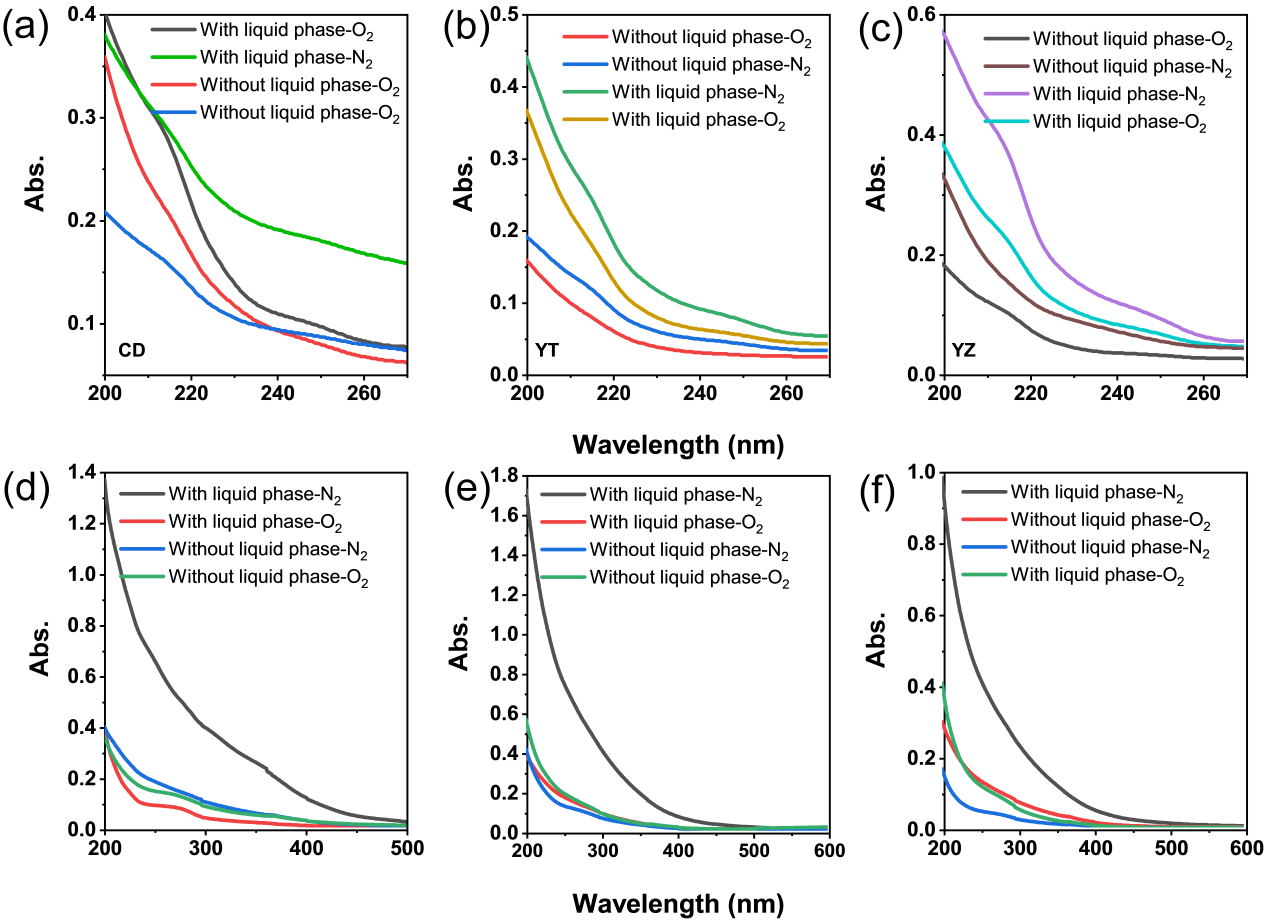


Figure S14. (a, b, c) UV-vis spectra of the deep-soil slurries and (d, e, f) topsoil slurries.





Figure S15. Relative frequencies of molecular properties for the original YT_0-20_ and samples after oxidation. (a, d) double bond equivalence (DBE), (b, e) the molecular weight (MW), and (c, f) aromatic index (AI). (g, h) Venn diagrams of the number of molecules identified by different pretreatments. (i) The relative abundance of seven assigned compound classes of DOM.


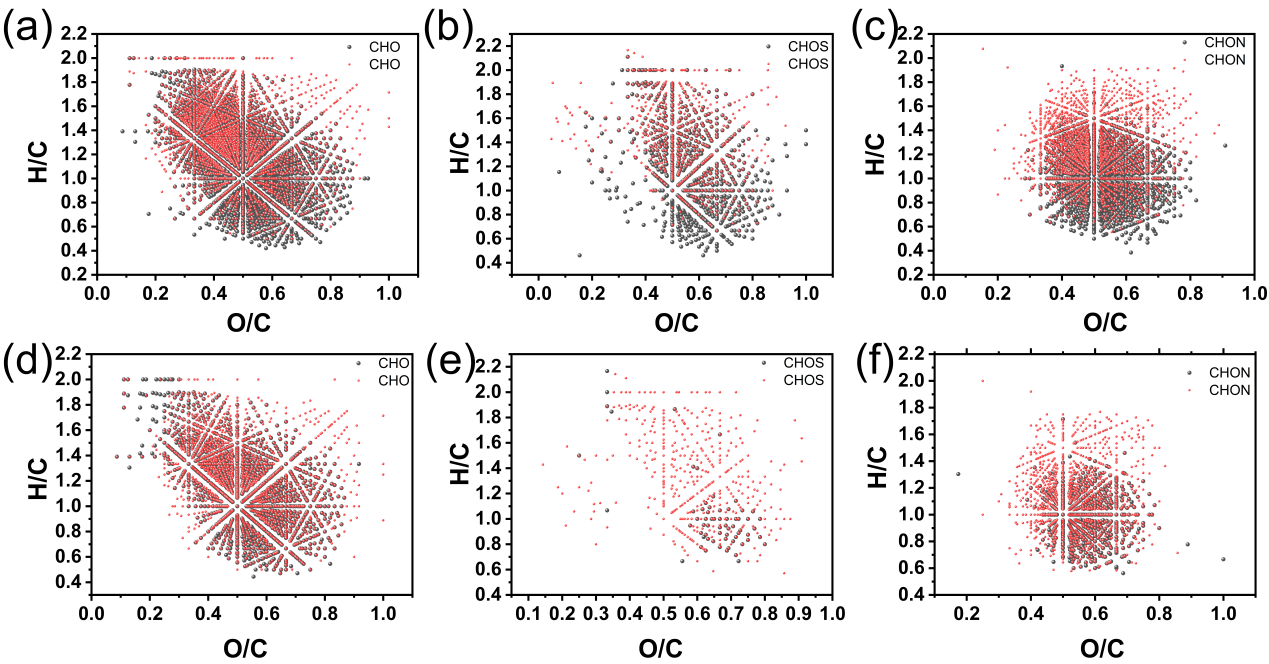


Figure S16. The different element composition van Krevelen diagrams of DOM before (black) and after (red) oxidation. (a, d) CHO, (b, e) CHOS, and (c,f) CHON (with or without the original aqueous phase, respectively).


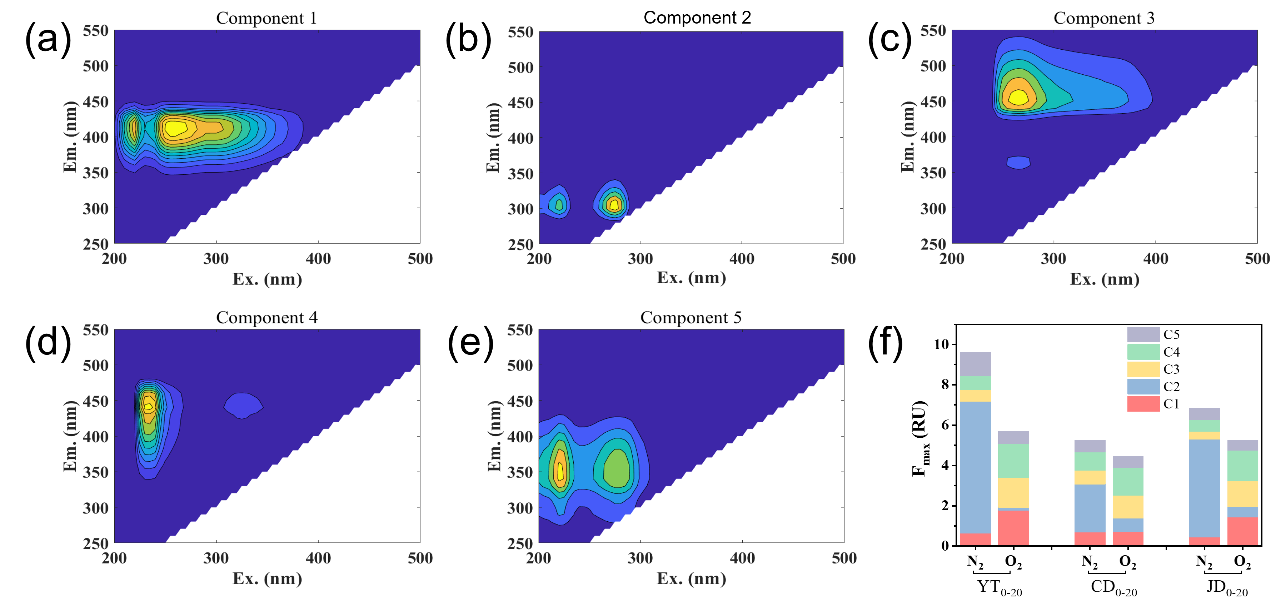


Figure S17. Excitation emission matrix contour plots of (a-e) the five fluorescent components (C1 to C5) identified by the PARAFAC analysis, and (f) the F_max_ of the five components in different treatments.





Figure S18. Relationships between the CO_2_ efflux concentration and (a) biological index (BIX), (b) fluorescence index (FI), (d) humification index (HIX), and (e) spectral slope ratio (*S_R_*). (c) HIX and (f) *S_R_* values of anoxic/oxc treatments of three paddy soil slurries.**References**

[1] W.C. Wang, D.Y. Huang, D.X. Wang, M.X. Tan, M.Y. Geng, C.Y. Zhu, N. Chen, D.M. Zhou, Extensive production of hydroxyl radicals during oxygenation of anoxic paddy soils: Implications to imidacloprid degradation, Chemosphere, 286 (2022) 131565.

[2] H. Tamura, K. Goto, Yotsuyan.T, M. Nagayama, Spectrophotometric determination of iron(II) with 1,10-phenanthroline in presence of large amounts of iron(III), Talanta, 21 (1974) 314-318.

[3] L.Y. Chen, L. Liu, S.Q. Qin, G.B. Yang, K. Fang, B. Zhu, Y. Kuzyakov, P.D. Chen, Y.P. Xu, Y.H. Yang, Regulation of priming effect by soil organic matter stability over a broad geographic scale, Nature Communications, 10 (2019).

[4] P. Rovira, V.R. Vallejo, Examination of thermal and acid hydrolysis procedures in characterization of soil organic matter, Communications in Soil Science and Plant Analysis, 31 (2000) 81-100.

[5] Q.L. Fu, M. Fujii, T. Riedel, Development and comparison of formula assignment algorithms for ultrahigh-resolution mass spectra of natural organic matter, Analytica Chimica Acta, 1125 (2020) 247-257.
